# Allogeneic Bone Marrow Mesenchymal Stem Cell Transplantation in Tooth Extractions Sites Ameliorates the Incidence of Osteonecrotic Jaw-Like Lesions in Zoledronic Acid-Treated Rats

**DOI:** 10.3390/jcm9061649

**Published:** 2020-05-31

**Authors:** Francisco Javier Rodríguez-Lozano, Ricardo Oñate-Sánchez, Mar Gonzálvez-García, Marta Vallés-Bergadá, Carlos M. Martínez, Beatriz Revilla-Nuin, Julia Guerrero-Gironés, Jose M. Moraleda, David García-Bernal

**Affiliations:** 1 Special Care in Dentistry-Gerodontology Unit, Department of Dermatology, Stomatology, Radiology and Physical Medicine, Morales Meseguer Hospital, Faculty of Medicine, University of Murcia, 30008 Murcia, Spain; reosan@um.es (R.O.-S.); juliaguerrero1@hotmail.com (J.G.-G.); 2 Research Group Cellular Therapy and Hematopoietic Transplant, Biomedical Research Institute, Virgen de la Arrixaca Clinical University Hospital, IMIB-Arrixaca, University of Murcia, Avenida Buenavista s/n, 30120 Murcia, Spain; jmoraled@um.es (J.M.M.); david.garcia23@um.es (D.G.-B.); 3 Faculty of Health Sciences, Catholic University of Murcia, 30107 Murcia, Spain; mdmgonzalvez@ucam.edu; 4 Dental and Maxillofacial Surgery Unit, Quirónsalud Torrevieja, Partida de la Loma, s/n, 03184 Torrevieja, Alicante, Spain; mvalles@clinicamartavalles.com; 5 Experimental Pathology Unit, Biomedical Research Institute of Murcia-Arrixaca, IMIB-Arrixaca, 30120 Murcia, Spain; cmmarti@um.es; 6 Genomics Unit, Biomedical Research Institute of Murcia-Arrixaca, IMIB-Arrixaca, 30120 Murcia, Spain; brevilla_nuin@yahoo.es; 7 Internal Medicine Department, Faculty of Medicine, University of Murcia, 30120 Murcia, Spain

**Keywords:** bone marrow mesenchymal stem cells, zoledronic acid, osteonecrosis, jaw, MRONJ

## Abstract

Medication-related osteonecrosis of the jaw (MRONJ) is defined as the exposed necrotic bone involving the maxillofacial structures in bisphosphonate treated patients, and the pathophysiology of this disease remains unclear. The aim of this study was to assess the effects of the allogeneic transplantation of bone marrow-derived mesenchymal stem cells (BM-MSCs) in a model of Wistar mice with induced MRONJ disease. BM-MSCs from five male Wistar rats were characterized and cultured on β-tricalcium phosphate (β-TCP) granules. Thirty female Wistar rats were injected intraperitoneally with zoledronic acid and afterwards upper jaw molars were extracted. The animals were randomized to receive: Group 1: 1 × 10^6^ BM-MSCs/β-TCP construct in the alveolar socket; and Group 2: Saline solution/β-TCP construct. A clinical and histological analysis was performed. Nested polymerase chain reaction (PCR) was assessed to verify the presence of transplanted male rat cells in the female recipient jaws. Clinical and histological findings evidenced that none of the animals in Group 1 exhibited uncovered sockets or bone exposure associated to MRONJ, whereas we detected 33% of MRONJ cases in Group 2. In addition, male rat cells were detected in the maxillae site four weeks after transplantation in the BM-MSCs-group. Allogeneic BM-MSCs in extractions sites ameliorates MRONJ incidence in zoledronic acid-treated rats compared to non-MSC treatments.

## 1. Introduction

In 1906, necrosis of jaw was described among those people working in the matchstick industry, an occupational disease provoked by inhalation of white phosphorus vapor and lack of the appropriate security measures. The incidence of these disorder, called “phossy” jaw almost disappeared after the ban on the use of phosphorus in matchsticks at the convention held in Bern (Switzerland) [1].

Nevertheless, a reappearance of this disease was noticed and reported in 2003, when osteonecrosis of the jaw was identified as an adverse side effect of pamidronate and zoledronate treatment [2]. These bisphosphonates—and other type of anti-resorptive drugs used for preventing osteoclast-mediated bone resorption—have been widely used to treat primary osteolytic bone pathologies such as Paget disease, osteoporosis, multiple myeloma and metastatic bone disease [3].

Medication-related osteonecrosis of the jaw (MRONJ) is defined as the exposed necrotic bone involving the maxillofacial structures that fails to heal after eight weeks. It has a great impact in the oral and maxillofacial clinical area and the pathophysiology of this disease remains unclear to date. Possible pathophysiologic mechanisms involved in MRONJ include the delay of bone remodeling [4,5], direct toxicity to the mucosal barrier [6,7], an altered angiogenesis that could lead to avascular necrosis [8,9], and an altered function of macrophages and T cells that would facilitate subsequent infections [10].

The aim of the treatment of patients at risk to development MRONJ should be based on prevention [11], being as once the disease is established, therapeutic interventions are not uniformly successful and have been controversial in recent years [12]. Lately, new regenerative medicine strategies such as cell therapy using mesenchymal stem cells (MSCs) for bone regeneration have arisen as a promising treatment [13,14,15,16].

MSCs are adult progenitor cells distributed in every organ and tissue with capacity to differentiate to adipocytes, osteoblasts and chondrocytes [17]. They have shown promising results in cell architecture repair, wound healing and recovery of local blood flow in damaged and ischemic tissues [18]. In addition, MSCs secrete multiple cytokines and trophic factors with immunomodulatory and anti-inflammatory properties [13,16], and also, they lack human leukocyte antigen (HLA)-II expression and a limited expression to HLA-I, that make them suitable for allogeneic transplantation. Remarkably, MSCs can be easily obtained under local anesthesia from various anatomic sites such as bone marrow, adipose tissue, dermis, neural tissue, periodontal ligament and dental pulp [19,20,21], all of which are reasonably non-invasive procedures. Furthermore, ex vivo culture and expansion is feasible and not difficult.

To date, few studies have tested the beneficial properties of MSCs for MRONJ prevention and treatment, including their systemic infusion [13,16,22] or local application [23]. Some of these studies in different animal models generated encouraging clinical results, but more research is needed prior to the clinical application of this therapeutic approaches. In this study, we aim to assess the effects of the allogeneic transplantation of bone marrow-derived-MSCs (BM-MSCs) in a proven model of Wistar mice with induced MRONJ disease.

## 2. Experimental Section

Animals used in this study were obtained from the Animal Facility Research Support Unit at the University of Murcia (Spain) (REGA ES300305440012). The Bioethics Committee at the University of Murcia (A1320141001) approved the experimental protocol. The entire study was conducted according to the European Union guidelines for animal experimentation (EU/63/2010).

In this quantitative and experimental research study, a total of 35 Wistar rats (30 female and 5 males; 250–300 g; 8–12 weeks old) were included. Rats were kept in clean cages (five animals per cage) with litter bedding and 12-h light cycle. Normal diet and water were provided ad libitum.

### 2.1. BM-MSCs Isolation, Characterization and Culture on Scaffolds

Donor healthy male Wistar rats (*n* = 5) were anesthetized and sterilized using intraperitoneal injection of sodium thiopental (50 mg/kg) using 75% ethanol for 20 min. After removing the femurs under sterile conditions, cells were flushed out with phosphate buffer saline (PBS) with penicillin/streptomycin. Bone marrow mononuclear cells were isolated by Ficoll density gradient centrifugation over Histopaque-1077 (Sigma-Aldrich, St. Louis, MO, USA), plated in 175 cm^2^ culture flask at 1.5 × 10^5^ cells/cm^2^ in DMEM low glucose (Gibco, Thermo Fischer Scientific, Waltham, MA, USA) supplemented with 10 fetal bovine serum (Gibco, Thermo Fischer Scientific, Waltham, MA, USA), 1% L-glutamine (Lonza, Basel, Switzerland), 100 U/mL penicillin and 100 μg/mL streptomycin (Lonza, Basel, Switzerland) (complete culture medium) and incubated at 37 °C and 5% CO_2_. BM-MSCs from passages 3 were used in all experiments.

Immunophenotype characterization of BM-MSCs were analyzed by flow cytometry using a FACSCanto flow cytometer (Beckton Dickinson, San Jose, CA, USA) after staining with fluorochrome-conjugated monoclonal antibodies specific for markers CD73 (clone 5F/B9, Beckton Dickinson, San Jose, CA, USA), CD90 (clone HIS51, eBioscience, San Diego, CA, USA), CD105 (clone 8A1, Abcam, Cambridge, UK), CD34 (clone ICO-115, Abcam, Cambridge, UK) and CD45 (clone OX1, eBioscience, San Diego, CA, USA).

A commercially available bone graft substitute (granules) was used: synthetic β-tricalcium phosphate (β-TCP) (Odoncer, Teknimed, L’Union, France) with size of 0.5–1.0 mm, 50% porosity and pore size between 100–1000 μm. This dimension was appropriate for the specific subcutaneous/intramuscular implantation. Under aseptic conditions in the laminar flow hood, the sterile β-TCP granules were pre-moistened in complete medium for 30–60 min. For cell seeding in the study group, BM-MSCs were detached from the culture flasks by trypsinization, centrifuged at 400 g and then re-suspended in complete culture medium.

To assess the continuing effect of β-TCP on the behavior of BM-MSCs in terms of cell adherence and growth, study periods of 24 h and 7 and 15 days were established. Then, BM-MSCs were directly seeded onto β-TCP granules at a density of 5 × 10^4^ cells/mL. In the control group, β-TCP granules were pre-moistened with complete culture medium without BM-MSCs. After 24 h, 7 and 15 days of culture, the cell-scaffold constructs were fixed with PBS and 3% glutaraldehyde in 0.1 M cacodylate buffer for 1.5 h at 4 °C. Then, they were rinsed again and dehydrated via a graded series of ethanol (30–90% v/v). Final drying was performed by the critical-point method (CPDO2 Balzers Union, Balzers, Liechtenstein). Before observation with a scanning electronic microscope (SEM) (JEOL-6100, Oxford Instruments, Abingdon, United Kingdom), samples were mounted on stubs and sputtered gold/palladium coated.

### 2.2. Experimental Design

Rats were considered as animal model for bisphosphonate-related osteonecrosis of the jaws due to is bigger size more suitable for manipulations, extractions and implant placement than mice [24,25]. All female animals (*n* = 30) received zoledronic acid (ZA) (Zometa^®^ 0.05 mg/mL (Teva Pharmaceutical Industries, Petaj Tikva, Israel)), at a dose of 0.1 mg/kg body weight. The rats were weighted before every experimental phase to properly dose the administered drugs, as well as for controlling weight gain or loss during the study. The medication was administered by intraperitoneal injection three times per week, for nine weeks in accordance with previous studies [23,26].

The rats were randomly assigned to the following groups:

Group 1 consisted of 15 female rats that received ZA + implantation of 1 × 10^6^ allogeneic BM-MSCs/β-TCP construct.

Group 2 (control) consisted of 15 female rats that received ZA + implantation of PBS/β-TCP construct.

Extractions of the three right upper molars in each animal were performed in the eighth week of treatment. One hour before the procedure, dipyrone was applied subcutaneously (160 mg/kg). The rats were weighed and then intraperitonially injected with 100 mg/kg of ketamine (Ketavet 100, Gellini Farmaceutici Spa, Peschira Borromea, Milan, Italy), in combination with 10 mg/kg of xylazine (Rompun, Bayer AG, Leverkusen, Germany) for general anesthesia. The three molars were dislocated and removed with infant forceps number 1. In addition to the exodontia, it was also carried out a bone cut (osteotomy) in the surgical cavity, to insert the scaffolds. For the fixation of the scaffold in the cleft, animals were treated with a buccal mucoperiosteal flap to cover the alveolar bone post-extractions. Light subperiosteal debridement and advancement of the buccal mucosa were performed. Subsequently, gingival borders were sutured with 6–0 Nylon thread and washed with polyvinylpyrrolidone-iodine, covering the defect without tension. Tramadol at a dose of 0.075 mg/kg body weight was administrated subcutaneously each day for the first 3 post-operative days.

### 2.3. Macroscopic Analysis

Changes in wound healing of tooth extraction site was performed at 4 weeks post-extraction, because of the faster healing period reported in rats compared to humans [27]. Clinical analyses were assessed by the senior investigator and a maxillofacial surgeon in a lighted room as previously published [28]. The following parameters were analyzed: exposed bone, level of wound healing, infection and degree of inflammation. A scale from 0–3 was used, 0 representing favorable healing and absence of clinical signs of osteonecrosis and 3 representing severe signs of impaired healing and osteonecrosis.

### 2.4. Histological Observation

Maxillary bone samples were fixed in 4% paraformaldehyde for 48 h at 4 °C. Specimens were decalcified with 10% ethylenediaminetetraacetic acid (EDTA) solution at room temperature (RT) for 4–6 weeks. Then, samples were dehydrated in an ascending series of ethanol solution and finally embedded in paraffin. Serial sections (3–4 μm) were obtained and stained with hematoxylin and eosin (HE) staining according to protocol. These pictures were recorded using a light microscope (Olympus, Tokyo, Japan).

Immunohistochemical analysis for osteocalcin expression was performed with an indirect-ABC immunohistochemical procedure was performed by using a commercial kit (Dako EnVision Flex (Agilent, Santa Clara, CA, USA). Briefly, after deparaffination, rehydration, demasking antigen and peroxidase blocking treatments, sections were incubated overnight with a polyclonal anti-osteocalcin antibody (Abcam, Cambridge, UK, dilution 1:200) and with the labeled polymer anti-rabbit for 20 min. Sections were revealed with 3–3′ diaminobencidine (DAB) and hematoxylin counterstained. Positive reaction was identified as a dark-brown precipitated, with an osteoblastic intracytoplasmic pattern.

For immunohistopathologic examination, the following parameters were taken into account: (1) degree of osteonecrosis as presence of 8–10 adjacent empty lacunae in alveolar bone, according with previously reports [29,30]; (2) inflammatory infiltrate; (3) degree of fibrosis; (4) neoformed bone; (5) neovascularization; (6) osteocalcin expression; and (7) presence of osteoclasts. Evaluation was performed in a semi-quantitative scale as follows: absence (value 0), mild (value 1), moderate (value 2), severe (value 3) and very severe (value 4); except for evaluation of neoformed bone that was as follows: absence (value 0), 5–25% (value 1), 25–50% (value 2) and >50% (value 3). Lastly, the presence of osteoclasts was represented as the average numbers of osteoclasts per 10-high power fields (×400).

### 2.5. Nested Polymerase Chain Reaction (PCR) Protocol for Y Chromosome-Specific DNA Detection in Rat Decalcified Jaw Samples

Nested polymerase chain reaction (PCR) was assessed to determine the presence of transplanted male rat cells in the female recipient jaws using the sex-determining region of the Y chromosome (SRY) as a quantification marker. DNA was extracted from formalin-fixed, paraffin-embedded rat jaws sections using QiAamp DNA FFPE tissue Kit (Qiagen, Hilden, Germany), following the manufacturer′s instructions. Then, xylene was added to four 5-μm-thick sections of FFPE samples to remove paraffin. Tissue was digested with proteinase K at 56 °C for 1 h. After washing, DNA was eluted with distilled water. The extracted DNA was quantified by absorbance at 260 nm and its purity was evaluated by the absorbance ratio at 260/280 nm with a NanoDrop-2000 spectrophotometer (Thermo Scientific, Waltham, MA, USA). Detection of rat transcribed testis specific protein (TSPY) gene, a Y-chromosome exclusive gene, was performed by two consecutive PCRs. The product of the first PCR was used as a template in the second PCR. Both PCRs were carried out by using 35 cycles of 94 °C for 30 s, 60 °C annealing temperature for 45 s and elongation at 72 °C for 40 s. Reactions were performed using 1-U/μL Taq DNA polymerase (Roche Diagnostics, Basel, Switzerland) using the supplier’s buffer (1×: 10 mM Tris-HCl, 1.5 mM MgCl_2_, 50 mM KCl pH 8.3), 0.2 mM mix dNTPs and 1 μM of each primer. The primer sequence was as follow: TSPY-Fwd: ATTCCGGGAACTGGTACTCC; and TSPY-Rev: AGGGGTACCCAATCTTCCAC. The final PCR product was run on an 2% agarose gel. Molecular weight marker 0.5 μg/lane of 100 bp DNA Ladder (Invitrogen, Molecular Probes, Eugene, CA, USA) was used.

### 2.6. Statistical Analysis

Data were analyzed using the Graph-Pad Prism (version 8.1.0, GraphPad Software, San Diego, CA, USA). One-way analysis of variance (ANOVA) followed by Tukey′s post hoc test for parametric values and Mann–Whitney U test for nonparametric values were used. Data are presented as the mean ± standard deviation. Differences were considered statistically significant when *p* < 0.05.

## 3. Results

### 3.1. Isolation, Characterization and Culture on Scaffold

After isolation, the average time required for cell adhesion was 48–72 h. Isolated cells evidenced a radiative or spindle shape and the polar growth under a microscope. Bone marrow cells contained heterogeneous cell population in shape, among which MSCs were no more than 50%. After one month of culture, a homogeneous population of stromal cells was evidenced.

**Figure 1:**
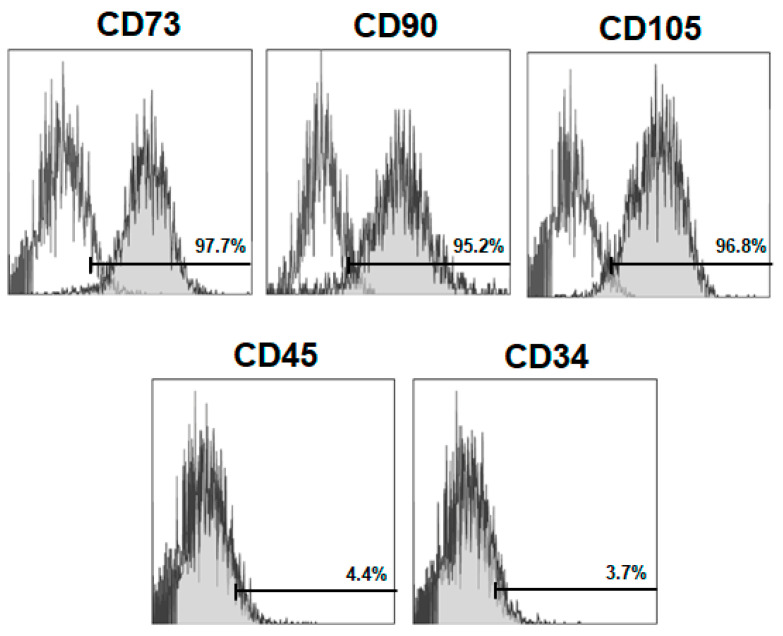
Rat bone marrow-derived-MSCs (BM-MSCs) express the typical mesenchymal stem cells (MSCs) markers CD73 (97.7%), CD90 (95.2%) and CD105 (96.8%), whereas expression of CD45 and CD34 were low or negative. Specific antibodies staining (light gray histograms) were compared with their corresponding control isotypes (white histograms). Values inside histograms represent percent of positive cells for each specific marker.

To verify the mesenchymal immunophenotype, BM-MSCs were characterized by flow cytometry. Cells expressed levels of specific MSC-surface markers, such as CD73, CD90 and CD105 and lacked the expression of the non-specific markers of MSCs (hematopoietic): CD34 and CD45 (Figure 1).

**Figure 2:**
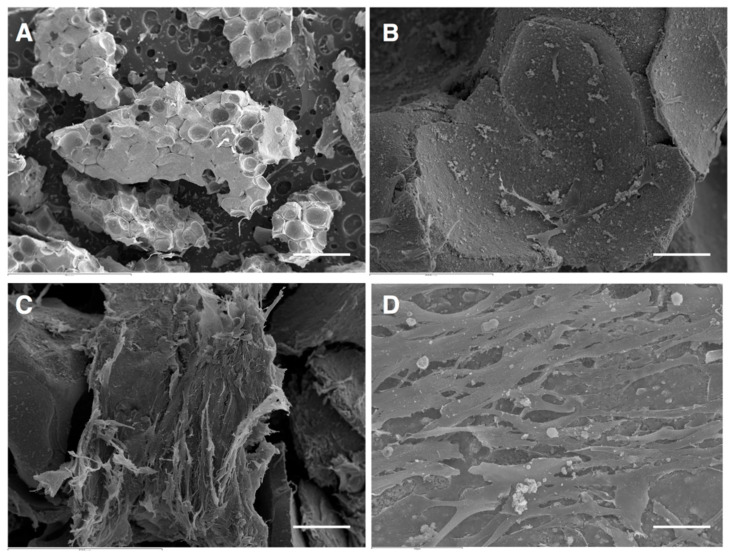
Scanning electron microscopic photomicrographs showing BM-MSC morphologic features and attachment and growth on β-tricalcium phosphate (β-TCP) scaffolds. (**A**) Morphology of β-TCP scaffolds without cells; (**B**) few cells were detected adhered to β-TCP granules after 24 h of culture; (**C**) abundant cells well adhered to β-TCP granules and active adhesive interactions with the scaffold surface was observed at 7 days; (**D**) multilayered cultures of BM-MSCs adhered to the β-TCP granules was observed at 14 days. Scale bar: 100 μM.

BM-MSCs adhesion and growth on β-TCP granules was performed by scanning electron microscopy (SEM) micrographs. After 24 h, few BM-MSCs were detected adhered to β-TCP granules. Importantly, at 7 days, it was observed that cell density was increased and covered the biomaterial, exhibiting multiple extensions that anchored the cells to the β-TCP granules. After 14 days of culture, multilayered cultures of BM-MSCs adhered to the biomaterial, were evidenced. Furthermore, irregular-shaped particles and numerous extracellular matrix components and were observed on the surface of the cells (Figure 2).

### 3.2. Clinical Visualization of MRONJ

**Figure 3:**
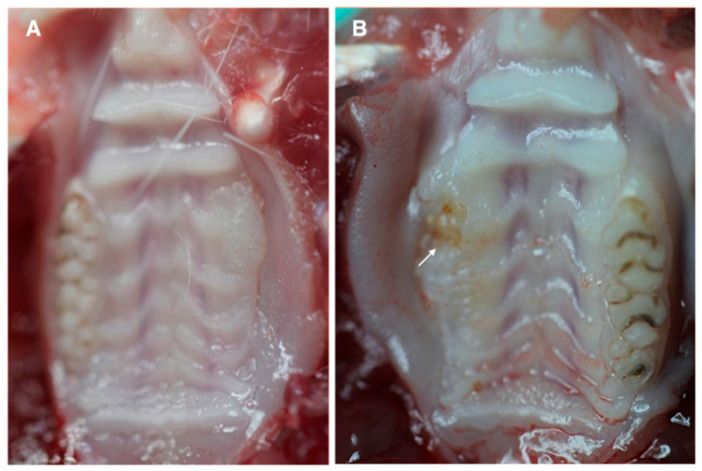
Tooth extraction sites in ZA-treated rat mandibles 4 weeks after being transplanted with (**A**) BM-MSC + β-TCP scaffold or (**B**) control saline + β-TCP scaffold. The last group displayed osteonecrotic lesions such as mucosal ulcerations and exposure of necrotic bone areas.

After 4 weeks of tooth extraction, none of the animals in Group 1 (i.e., rats that received ZA + implantation of 1 × 10^6^ allogeneic BM-MSCs/β-TCP constructs) evidenced bone exposure or uncovered sockets associated to MRONJ (Figure 3A). In contrast, 33% of the animals in Group 2 (i.e., rats that received ZA + implantation of saline/β-TCP constructs) showed osteonecrotic jaw-like lesions such as mucosal ulcerations at the teeth extraction site and frequent exposure of necrotic bone areas (Figure 3B).

### 3.3. Histological Analysis

**Figure 4:**
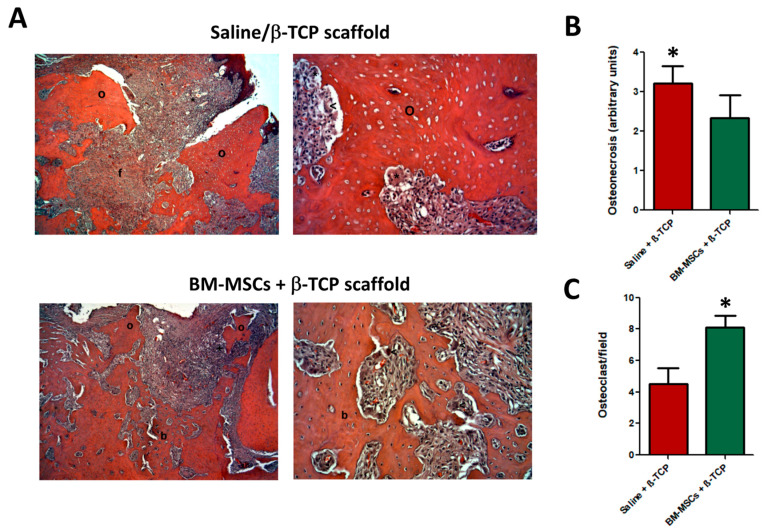
Representative images of HE staining of maxillary bone after induction of osteonecrosis in control animals transplanted only with saline + β-TCP scaffold, (**A**, top images) and those treated with BM-MSCs + β-TCP scaffold (**A**, bottom images) 4 weeks after treatment. Left images magnification: × 100; Right images magnification: ×400; (**B**) control animals displayed a higher degree of osteonecrosis than BM-MSC-treated counterparts (* *p* < 0.05) whereas osteoclast number per high-power field at 400× was significantly augmented in (**C**) the BM-MSC-treated group (* *p* < 0.05). Symbols: o: osteonecrotic areas; f: fibrotic areas; b: bone neoformation.

Microscopic examination of maxillary bone samples after HE staining revealed that animals of the control group treated only with the β-TCP scaffolds (i.e., w/o BM-MSCs) displayed typical histopathologic signs of osteonecrosis, i.e., formation of granulation tissue, inflammatory cell infiltrates, fibrosis and sequestra (Figure 4A, top images). Conversely, BM-MSC + β-TCP scaffold treated-group of animals showed new bone formation areas and a concomitant substantially reduced degree of osteonecrosis 4 weeks after BM-MSC transplantation (Figure 4A, bottom images).

Using a semi-quantitative scale for histological analysis, we showed that control animals displayed a higher degree of osteonecrosis than BM-MSC-treated counterparts (*p* < 0.05) (Figure 4B). Importantly, osteoclasts number, i.e., bone cell population that mediates bone resorption, was significantly increased in the BM-MSCs-treated group samples (*p* < 0.05), while the necrotic zone of the alveolar bone in the control group harbored lower osteoclast density (Figure 4C).

**Figure 5:**
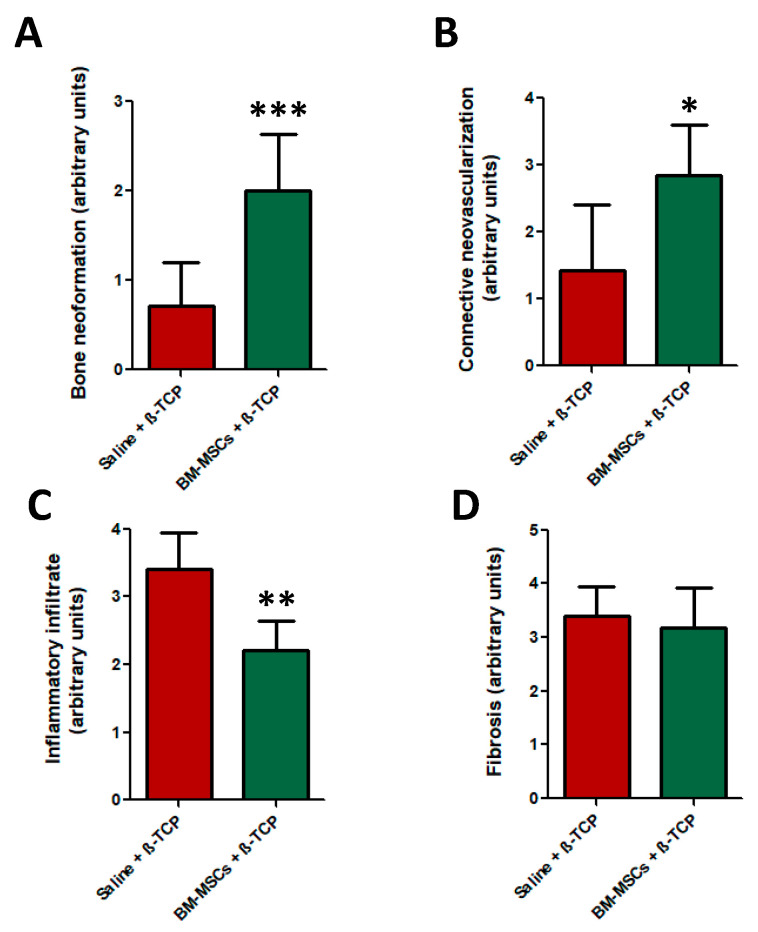
Evaluation of the effect of BM-MSC mediated bone neoformation, connective tissue neovascularization, inflammatory infiltrate and degree of fibrosis. (**A**) Maxillary bone of BM-MSC + β-TCP-treated animals showed a significant degree of bone neoformation; (**B**) connective tissue neovascularization and (**C**) significant lower inflammatory infiltrate, * *p* < 0.05, ** *p* < 0.01 and *** *p* < 0.001, respectively; (**D**) however, the degree of fibrosis was not significantly different between both experimental groups.

Histomorphometrical analysis demonstrated that only the BM-MSC-treated group showed a significant increased bone neoformation compared to control bone samples (*p* < 0.001) (Figure 5A). This new tissue contained abundant osteocytes and osteoblast cells in its lining surface (Figure 4A, bottom images). By contrast, the presence of necrotic bone or sequestra with loss of osteocytes from their lacunae was evident in control bone samples (Figure 4A, top images).

In addition, the effects of MSC-therapy on neovascularization of the connective tissue of tooth extraction sockets, degree of inflammatory infiltrate and degree of fibrosis were also analyzed. BM-MSCs-group samples displayed a significant increased number of blood vessels and vessel surface area compared to the control group (Figure 5B), as well as lower amounts of inflammatory cell infiltrates (*p* < 0.01). Regarding the degree of fibrosis, no significant differences between both experimental groups were detected (Figure 5D).

**Figure 6:**
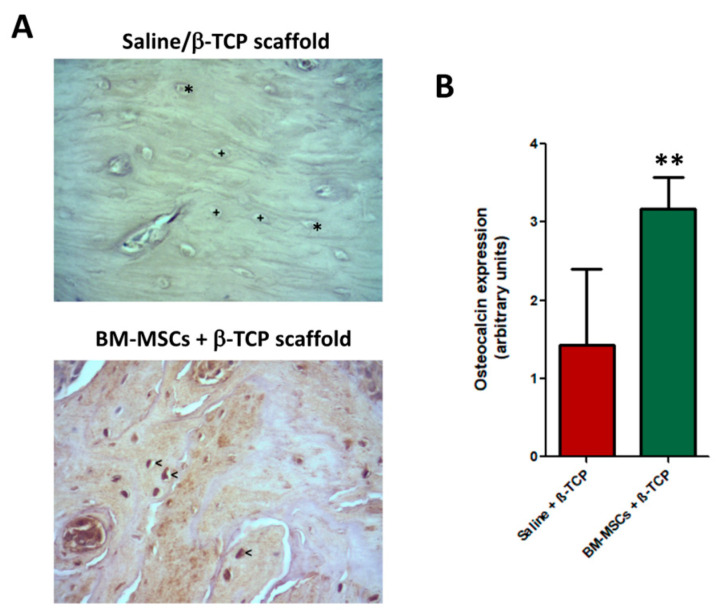
Representative images of immunohistochemical expression of osteocalcin of maxillary bone after induction of osteonecrosis in control animals transplanted only with saline + β-TCP scaffold, (**A**, top image) and those treated with BM-MSCs + β-TCP scaffold (**A**, bottom image) 4 weeks after treatment. While in control animals the empty lacunae (+) and osteocytes (*) are negative, osteocytes from BM-MSC-treated bone strongly expressed osteocalcin (<). ABC immunohistochemical procedure anti-osteocalcin were performed. Magnification: ×400; (**B**) number of osteocalcin positive cells were significantly more abundant, ** *p* < 0.01.

Regarding osteocalcin expression, BM-MSCs-treated group showed an evident higher expression of osteocalcin positive cells compared to control group (*p* < 0.001), thus evidencing the higher degree of bone neoformation previously addressed (Figure 6A–C).

### 3.4. Y-Chromosome Detection

**Figure 7:**
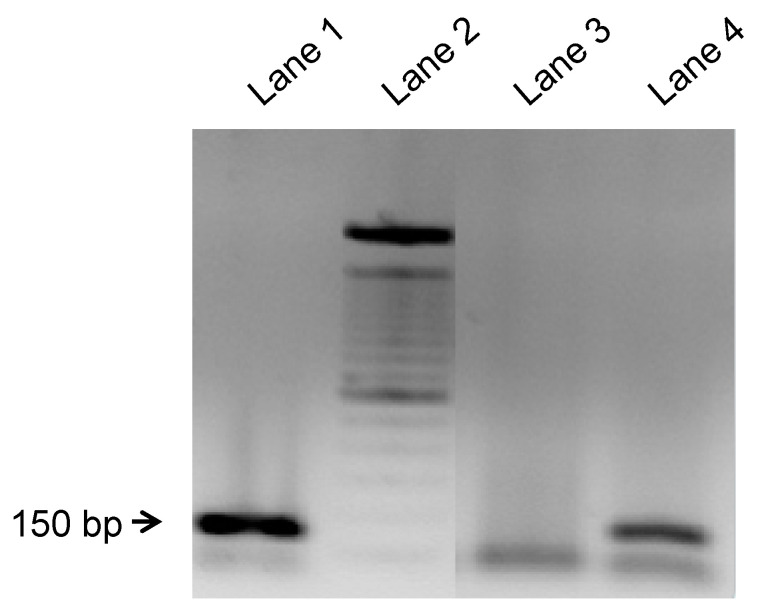
Representative analysis of nested polymerase chain reaction (PCR) for defining the presence of transplanted male rat BM-MSCs in the female recipient maxillary using the sex-determining region of the Y chromosome as a quantification marker. PCR amplification products were 150 bp. Lane 1: positive control (liver cells from male rats); Lane 2: 100 bp DNA ladder; Lane 3: maxillary cells from female rats transplanted with saline + β-TCP scaffold; Lane 4: maxillary cells from female rats transplanted with BM-MSCs + β-TCP scaffold.

Finally, bone marrow stromal cells, that were initially isolated from male rats, were detected in the maxillary site of transplanted female rats 4 weeks after transplantation in the BM-MSCs-treated group by detecting the sex-determining region of the Y-chromosome, while, as expected, there were no detected cells in the saline + β-TCP group (Figure 7).

## 4. Discussion

The prevention or treatment of MRONJ disease in patients receiving high doses of intravenous anti-resorptive drugs are still an unmet clinical need for maxillofacial surgeons. The estimated incidence of MRONJ in patients treated with intravenous ZA or other bisphosphonates for their different clinical applications ranges 0.8–12% [31], but evidence that explains its etiology remain inconclusive. Several therapeutic and preventive protocols for MRONJ have been proposed including conservative measures such as 0.12% chlorhexidine mouthwashes and antibiotics during the initial stages or the surgical approach that includes resection and reconstruction of the maxilla, aiming to prevent infections and/or inflammation and to decrease the stage of the disease. Other therapeutic alternatives have also been developed, including laser, ozone therapy, teriparatide, hyperbaric oxygen therapy, use of autologous platelet concentrates or MSC-based therapies [15,32]. However, BM-MSCs have not yet been proposed as an effective preventive treatment. In our study, we demonstrated that transplantation of allogeneic BM-MSCs in combination with β-TCP scaffolds in tooth extractions sites significantly ameliorates MRONJ in ZA-treated rats compared to those transplanted with only the scaffold.

To date, dental extractions were considered to be the most common risk factor for developing MRONJ and patients with other concomitant oral inflammatory diseases (e.g., periodontitis or dental abscesses) are at increased risk for developing MRONJ [32]. Previous experimental studies of MRONJ suggest that inflammation and oral infections contribute to its pathogenesis [33]. In this regard, there is increasing evidence that cell–cell interactions between MSCs and different immune cell populations, i.e., T cells, B cells, macrophages or dendritic cells, as well as MSC secretion of distinct immunomodulatory molecules or trophic factors, are likely to play role in the beneficial effects of MSC in inflammation-related diseases [34,35].

Previous reports have utilized dogs, mice and minipigs as animal models in MRONJ. However, most studies have utilized rats because they are a clinically relevant, inexpensive and easily replicable animal model with a high frequency MRONJ development after bisphosphonates treatment [23,24,26,36,37]. Furthermore, there are animal models able to develop MRONJ with and without tooth extraction. In these studies, histological examination in the animals injected with bisphosphonates which underwent dental extractions showed wide osteonecrotic areas; however, animals injected with bisphosphonates without tooth extraction only displayed periodontal inflammation [38]. Hence, unilateral extraction of three right molars were performed to trigger MRONJ development, where invasive dental procedures are the number one precipitating the main event for osteonecrosis appearance in bisphosphonate-treated human patients [39].

Autologous BM-MSCs have been utilized in patients with MRONJ lesions after necrosectomy, although the number of studies is very limited [14,15,40]. However, no reports were conducted to analyze the role of allogeneic BM-MSCs transplants to reduce the pathologic consequences of MRONJ. Although bone marrow harvesting is a non-invasive procedure, the medical conditions of the patients (i.e., bone fragility) could make obtaining autologous cells difficult, so we used allogeneic BM-MSCs.

In this study, we observed that animals with ongoing MRONJ and treated only with the β-TCP scaffolds (i.e., w/o BM-MSCs) showed evident histopathologic signs of osteonecrosis that were characterized by the formation of granulation tissue, an abundant inflammatory cell infiltrate, a decreased bone neoformation after tooth extraction, appearance of fibrotic tissue and low connective tissue neovascularization. Conversely, when these β-TCP scaffolds were cellularized with BM-MSCs, these cells were able to significantly reverse some of these osteonecrotic-related histopathologic events. Conspicuously, our results evidenced that necrotic zone of the alveolar bone in control animals was often located adjacent to inflammatory cells infiltrates. In this regard, some authors have also reported a correlation between necrosis and inflammation in MRONJ [13]. Although BM-MSC transplantation did not ameliorate the degree of fibrosis, BM-MSCs decreased the inflammatory infiltrate level, increased bone neoformation and neovascularization and importantly, gave rise to the presence of an abundant number of osteoblasts (osteocalcin positive cells) and osteoclasts, a histopathologic finding closely related with an active bone remodeling process [41]. In the same line with our results, Mergoni et al. [42] concluded that high expression of osteocalcin is associated with an intense osteoblast activity and bone healing.

Taken together, different cell therapy approaches using mesenchymal stem cells can offer effective and safe therapeutic alternatives for preventing MRONJ development in all those patients who must be treated with anti-resorptive drugs for conditions such as osteoporosis or other metabolic/neoplastic bone diseases.

## 5. Conclusions

In summary, allogeneic BM-MSCs implanted in extractions sites ameliorates MRONJ incidence in zoledronic acid-treated rats. Further studies are necessary to confirm the rationale for in vivo cell therapy using MSCs to prevent or treat MRONJ disease in human patients.
